# Trends and Challenges of Surgical Site Infections Burden in Croatia: A Nationwide Comparative Analysis of Two Point Prevalence Surveys (2017–2023)

**DOI:** 10.3390/life16020239

**Published:** 2026-02-02

**Authors:** Ana Gverić Grginić, Zrinka Bošnjak, Alen Babacanli, Zoran Herljević, Mislav Peras, Ivana Ferenčak, Igor Pelaić, Lana Videc Penavić, Ana Budimir

**Affiliations:** 1 Antimicrobial Resistance Prevention Laboratory Diagnostics Unit, Division for Microbiology, Croatian Institute for Public Health, 10000 Zagreb, Croatia; 2 Department of Clinical and Molecular Microbiology, University Hospital Centre Zagreb, 10000 Zagreb, Croatia; zrinkabosnjak@gmail.com (Z.B.); zoranhe@gmail.com (Z.H.); anchie.budimir@gmail.com (A.B.); 3 School of Medicine, University of Zagreb, 10000 Zagreb, Croatia; 4 Croatian Medical Chamber, 10000 Zagreb, Croatia; babacanli@gmail.com; 5 Department of Microbiology, Public Health Institute Zagreb County, 10000 Zagreb, Croatia; mislav.peras@gmail.com; 6 Department of Genomics, Division for Microbiology, Croatian Institute for Public Health, 10000 Zagreb, Croatia; ivana.ferencak@hzjz.hr; 7 Institute of Emergency Medicine Zagreb County, 10000 Zagreb, Croatia; igor.pelaic@gmail.com; 8 Reanimation and Intensive Care, Department of Anaesthesiology, University Hospital Centre Sestre Milosrdnice, 10000 Zagreb, Croatia; lanavidec@yahoo.com

**Keywords:** surgery, infections, prevention, surveillance

## Abstract

Background: Surgical site infections (SSIs) are among the most frequent healthcare-associated infections (HCAIs) worldwide. Changes in the functioning of healthcare systems may affect the implementation of SSIs prevention practices, with consequent alterations in the occurrence of HCAIs. The main aims of our study were to analyze specific SSIs prevalence and proportions together with overall HCAIs prevalence in acute care hospitals (ACHs) before and after the COVID-19 pandemic. Additional aims were to identify bacterial causative agents, the use of perioperative antibiotic prophylaxis (PAP), related structural and process quality indicators, and to determine trends between two periods. Methods: The National Reference Centre for HCAIs (University Hospital Centre Zagreb) conducted point prevalence surveys in May 2017 and May 2023 in ACHs throughout Croatia, using the technical protocol developed by the European Centre for Disease Prevention and Control (ECDC). Results: The prevalence of HCAIs in ACHs in Croatia rose from 5.3% (95% CI 4.8–5.7) in 2017 to 7.2% (95% CI 6.6–7.8) in 2023 (*p* = 9.93 × 10^−14^). This trend was paralleled with the rising of the HCAIs prevalence in surgical departments from 5.1% to 6.7% (*p* = 0.0099). The prevalence of overall SSIs across ACHs increased from 0.9% (95% CI 0.7–1.1) in 2017 to 1.2% (95% CI 1.0–1.5) in 2023 (OR 1.36 (1.03–1.80), *p* = 0.032. While the prevalence of superficial incisional SSIs significantly decreased (OR 0.53 (0.30–0.95), *p* = 0.028), the share of deep-seated SSIs (deep incisional and organ/space SSIs) among classified SSIs shifted from 48/92 to 77/96; odds ratio (OR) 2.09 (95% CI 1.45–3.01). In 2017, Gram-positive cocci were the most frequently isolated bacterial causative agents (44.6%). By 2023, this shifted, with Enterobacterales species comprising most isolates (42.2%). In 2023, significantly a higher proportion of patients received PAP (χ^2^ = 25.419, df = 1, *p*  < 0.5). An increase in the positive trend of alcohol-based hand rub antiseptics use in surgical departments (+15.7 L/patient-days, *p* < 0.001) contrasted with a decrease in infection prevention and control (IPC) nurses and medical doctors per hospital (−0.5, *p* = 0.041/−0.5, *p* = 0.003). Conclusions: Findings of the two point prevalence surveys over time indicate the changes in trends in surgical site infections burden, and highlight the need for the implementation and strengthening of preventive measures with the focus on targeted prevention of deep-seated infections.

## 1. Introduction

Healthcare-associated infections (HCAIs) are a major burden of modern healthcare systems worldwide, resulting in additional morbidity and mortality attributable to these, often preventable, diseases. Among them, surgical site infections (SSIs) represent a substantial proportion [1,2,3]. Furthermore, besides compromised patient safety during healthcare and poorer outcomes of initial surgical interventions, SSIs significantly contribute to escalating financial costs, due to a prolonged hospital length of stay (LOS), the need for additional diagnostic and therapeutic interventions requiring hospital readmissions, and an increase in disability-life years (DALYs) and years of life lost (YLL) [4,5,6]. In recent decades, a higher volume of surgical procedures, particularly in the aging population, combined with the greater complexity of advanced surgical and intensive care techniques, have created higher risks of acquiring surgical site infections [7]. The alarming increase in multidrug-resistant (MDR) bacteria causing HCAIs poses an added challenge in the management of SSIs, further complicating treatments and potentially leading to poorer therapeutic outcomes [8].

Globally, the prevalence rate of overall HCAIs shows a trend of annual increase, with differences depending on World Health Organization (WHO) geographic regions, the countries’ incomes, patients’ age and sex, the level of hospitals and type of department [9]. Research by Raooffi et al. has determined that surgical site and wound infections are the most frequent type of infections among HCAIs worldwide, with pooled proportions among HCAIs of roughly 0.34 and 0.26, respectively [9]. The ECDCs latest annual epidemiological reports of SSIs in European Union/European Economic Area (EU/EEA) countries show that the incidence of SSIs per 100 operations was 0.6% in laminectomies, whereas the incidence in open colon surgeries was 9.6%. These data confirm that SSIs depend on surgical procedures, with the lowest incidence in ultra-clean surgery, such as knee and hip replacements, and the highest incidence in open colon surgery [10]. The interplay of various risk factors contributes to the development of SSIs, including the patient’s preoperative clinical status, the invasiveness and duration of the surgical procedure, the surgical technique employed and the adherence to evidence-based infection control practices in the pre-, intra- and post-operative period [11]. Therefore, prevention strategies and effective risk reduction management protocols need to encompass an extensive spectrum of potential risk factors for the onset of disease [12,13]. The main tool for the assessment of prevention practice indicators and outcomes is regular surveillance; its results represent the basis for planning prevention procedures and interventions. In EU/EEA countries, the ECDC conducts point prevalence studies of HCAIs and antimicrobial use every five years, following a standardized protocol and data collection methodology, with the first study conducted in 2011–2012 and the latest in 2022–2023 [14]. From 2020 to 2022, healthcare systems were affected and overloaded by the COVID-19 pandemic and were experiencing momentous changes to their standard roles and procedures. Hospitals had increased bed occupancies due to increasing numbers of patients with COVID-19 and had limited capacities for non-urgent patients and surgeries. In addition, healthcare workers of various specialties were reassigned to COVID-19 departments. A reduction in access to surgeries due to these factors, together with the lockdown, influenced the epidemiology of SSIs. Whereas some studies reported a reduction in SSIs after the COVID-19 pandemic, reflecting the adaptation to enhanced hygienic protocols, other studies reported stable rates, or even an increase, depending on surgical procedure, underlying disease and delay of surgery [15,16,17].

The aims of our study were to compare the burden of surgical site infections nationwide in order to yield critical insights into the epidemiological dynamics of these infections between 2017 and 2023, focusing on several key parameters: trends in prevalence and proportion rates depending on the categorization of the SSIs, the identification of the causative pathogens, the prevalence of SSIs across various surgical procedures, the evaluation of key quality indicators related to surgical site infection prevention, and antibiotic consumption in surgery, with an emphasis on the perioperative antibiotic prophylaxis (PAP).

## 2. Materials and Methods

During May 2017 and May 2023, national multicentre cross-sectional prevalence surveys were conducted in acute care hospitals in Croatia.

Both point prevalence surveys were part of the ECDC point prevalence surveys of HCAIs and antimicrobial use in acute care hospitals 2016–2017 (HR-PPS-1) and 2022–2023 (HR-PPS-2). Surveys were conducted according to ECDC protocols, v5.1 and v6.1, respectively [14,18]. All wards within each acute care hospital, except emergency units, were included in the surveys. The duration of the surveys was one day per department. The entire duration of the surveys was limited to three weeks.

A standardized form provided in ECDC PPS protocols was used for data collection. The Croatian Reference Centre for Healthcare-Associated Infections (RC for HCAIs) in University Hospital Centre Zagreb conducted and coordinated the surveys. All data were collected in RC for HCAIs. Coordination included previous one-day in-person training seminars for personnel who collected the data in both surveys. Data collecting personnel were infection prevention and control (IPC) team members in the hospitals which were included in the surveys. IPC teams in Croatia consist of one medical doctor and nurse(s) educated in IPC practices. Education is obligatory according to national regulation and it consists of participating in a postgraduate course in the School of Medicine, University of Zagreb. Medical doctors in IPC teams can have different specialties (clinical microbiology, infectious diseases or epidemiology). During the survey period, consultation with the coordinator in RC for HCAIs was enabled via a call centre.

Data were collected directly on wards from medical records—with the attending medical doctor on the ward—registered in the standard universal ECDC form and subsequently entered in HELICSWin.Net software (Version 2.3) by a trained IPC team member [19]. Aggregated and anonymized data were sent to RC for HCAIs, where they were validated, aggregated, transferred into the TESSy format and uploaded to TESSy (Version 1.4) (EpiPulse) [19]. Collected data included structure and infection prevention and control performance (IPC) indicators within acute care hospitals on both hospital and surgical department level. On the hospital level, data included the number of hospital beds, the number of specialized IPC healthcare-workers (IPC doctors and nurses separately), their ratio per 250 beds, the use of hand hygiene antiseptics (L/pt.-days), and participation in surgical site infections surveillance networks. On the surgical department level, data included the number of beds, the number of hospitalized patients during surveys in order to determine surgical bed occupancy, the number of patients with HCAIs, the number of patients having SSIs along with the registration of SSIs classification regarding the SSIs tissue depth invasiveness (SSI-S superficial incisional, SSI-D deep incisional, SSI-O organ/space, SSI-Nos category not specified/unknown), microbiological analyses of agents causing SSIs, patients who had received antimicrobial therapy in the duration of the study, and its indication. The ECDC case definitions for site-specific HCAIs diagnosis were used according to the protocol [18,19]. Numbers of patient-days in years before the surveys were conducted were extracted from the Croatian Health Statistics Yearbook 2017 and 2023 [20,21]. If applicable, additional data were collected on the type of the operative procedure undertaken and entered in the data collection form. Centres for Disease Control and Prevention (CDC) National Healthcare Safety Network (CDC/NHSN) classifications and codes were used in the classification of surgical site infections’ invasiveness and operative procedure codes [22]. According to NHSN definitions, surgery is defined as a procedure performed primarily for therapeutic reasons where an incision is made (not just a needle puncture), with a breach of mucosa and/or skin, not necessarily in the operating theatre. NHSN surgery codes were used as proxy/indicators for major surgical procedures. Examples of non-NHSN surgery/minimally invasive surgical procedures used were as follows: obstetrical procedures: peri-delivery/labor (one or more), dental extraction, transurethral resection of prostate, incision and drainage of abscess with secondary closure, any diabetic forefoot amputation with healing by secondary intention, any other operation where healing is by secondary intention, tonsillectomy, application of external fixator/Olizarov, extraventricular drain, hysteroscopic removal of fibroids, evacuation of retained products of conception [18,22].

Over the course of both surveys, all patients who met the ECDC case definitions for HCAIs were included in the survey, regardless of whether the origin of HCAI was related to their current hospitalization or HCAI was acquired in another acute care hospital in Croatia. Patients whose healthcare-associated infections originated from long-term, chronic healthcare or rehabilitation institutions were excluded from the surveys. In addition to the HCAIs type, availability of microbiological analysis, identified HCAIs microbial causative agent, and antibiotic consumption together with the indication for administration were registered.

The prevalence (with confidence interval) of overall HCAIs was reported as the percentage of patients with at least one HCAI over the total number of patients participating in the surveys. The prevalence of HCAIs associated with surgical departments was reported as the percentage of patients with HCAIs over the number of patients hospitalized in surgical departments. The prevalence of SSIs, overall and categorized by affected tissue depth, was reported as the percentage of SSIs among the total number of patients in the survey. The relative frequencies for HCAIs type, SSIs categories, and microbiological findings were reported as the percentage with the total number of HCAIs or total number of SSIs as the denominator. The prevalence of antibiotic consumption and the prevalence of its indications were reported as the percentage of patients receiving at least one antibiotic agent in a specific indication category (treatment intention or surgical prophylaxis).

The obtained data are presented in tabular and graphical forms. Continuous variables (e.g., the number of hospital beds, the number of beds in surgical departments, the number of patients hospitalized in surgical departments and the number of IPC healthcare-workers) are summarized as means, whereas categorical variables are reported as counts and percentages. Differences in proportions were assessed using Pearson’s χ^2^ test or Fisher’s exact test, as appropriate; paired comparisons used the paired t-test or the Wilcoxon Signed-Rank Test. Associations between continuous/ordinal variables were evaluated with Spearman’s rank correlation coefficient (ρ). Two-sided *p*  <  0.05 was considered statistically significant. Statistical analyses were performed in R (version 4.5.1; R Foundation for Statistical Computing, Vienna, Austria). All tests were two-sided with α  =  0.05 [23].

## 3. Results

### 3.1. Hospitals Participating in the Surveys

A total of 32 (32/34, 94.1%) acute care hospitals were included in the HR-PPS-1 survey in 2017. Thirty hospitals had surgical unit specialties (30/32, 93.7%). In 2023, 31/32 (96.8%) acute care hospitals were included in the HR-PPS-2 survey, with 25 hospitals having surgical departments (25/31, 80.6%). The difference in overall numbers of acute care hospitals and surgical departments eligible for inclusion was lower in the second survey because administrative, functional and structural mergers/restructuring of the acute care hospital network in Croatia were implemented in 2018. The proportion of acute care hospitals participating in the surveys was high in both periods, having an optimal sample representativeness for the ECDC PPS studies as defined by the ECDC technical protocol (recommended sample size achieved; inclusion of ≥ 75% of all acute care hospitals or ≥ 75% occupied acute care hospital beds in the country). Therefore, the results provide a reliable basis for the analyses presented in this study.

### 3.2. Hospital and Surgical Department Structure Indicators

The mean number of hospital beds across all ACHs was lower in 2023 than in 2017, as presented in Table 1. By contrast, the mean number of hospital beds specifically in surgical departments increased over time. Despite later finding, bed-occupancy rate and the number of patient-days in surgical departments decreased from 2017 to 2023. Spearman’s rank correlation coefficient (ρ = −0.012; S = 85.012; *p*  =  0.9774) showed no association between the mean numbers of hospital and surgical-department beds and the prevalence of HCAIs or SSIs.

### 3.3. Prevention Structure and Process Indicators

Prevention indicators, as shown in Table 2, moved in mixed directions between 2017 and 2023; while alcohol-based hand rub (AHR) consumption in surgical departments increased significantly, in contrast, the absolute number of IPC nurses and IPC medical doctors per hospital significantly decreased. Participation of ACHs in the SSI surveillance networks declined (−9 percentage points/pp), but did not show a statistically significant difference. Wilcoxon Signed-Rank Test for comparison of eligible prevention indicators (IPC healthcare-workers’ number, and ratio per 250 beds, consumption of alcohol-based hand rub in surgical departments), and McNemar’s test for binary variable (participation in surveillance networks; 1 for participation, 0 for non-participation) was conducted. Paired analysis included 29 paired ACHs in 2017 and in 2023.

### 3.4. Healthcare-Associated Infections on Hospital Level and on Surgical Department Level

As shown in Table 3, the overall prevalence of healthcare-associated infections (HCAIs) in acute care hospitals was 5.3% (95% CI 4.8–5.7) in 2017. In surgical departments, (28.5% of the surveyed patients) and HCAI prevalence was 5.1%. Comparing 2017 with 2023, HCAI prevalence increased both at the hospital level (5.3% → 7.2%) and at the surgical departments’ level (5.1% → 6.7%). Pearson’s Chi-squared test for proportions indicated a highly significant increase (χ^2^ = 55.38; *p* = 9.93 × 10^−14^) of HCAIs in all ACHs, together with a statistically significant increase in surgical departments, despite fewer patient-days in surgical departments in 2023.

#### Healthcare-Associated Infections in Specific Surgical Specialties

**Figure 1:**
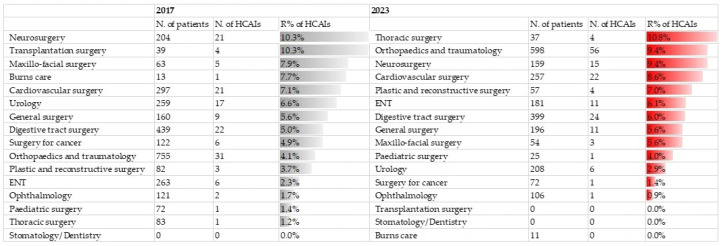
Prevalence of HCAIs in different surgical departments. N—number; R—relative frequency; HCAIs—healthcare-associated infections.

The majority of surgical patients were hospitalized in orthopaedics and traumatology, digestive tract surgery and cardiovascular surgery departments in both surveyed periods, as presented in Figure 1. The number and proportion of patients hospitalized in these departments did not differ between the two time periods. Data from the 2017 survey show that most healthcare-associated infections occurred in transplantation surgery, neurosurgery and maxillo-facial surgery, despite fewer inpatients in these specialties. In the second study period, most HCAIs were registered in thoracic surgery, orthopaedics traumatology and neurosurgery, respectively.

### 3.5. Surgical Site Infections

The proportion of site-specific HCAIs among patients hospitalized in surgical departments shows that surgical site infections were the third most common site-specific HCAIs, with a frequency of 15.8% and 14.9% in 2017 and 2023, respectively. In contrast to pneumonia, bloodstream infections and *Clostridioides difficile* infections, SSIs, together with urinary tract infection and catheter-related infections, showed a decreased proportion among all HCAIs. No statistically significant differences were determined in the distribution of individual site-specific infection types between 2017 and 2023, as presented in Table 4.

Table 5 summarizes the trends of burden in specific SSIs categories by tissue depth. Overall, SSI prevalence increased from 0.9% (95% CI 0.7–1.1) in 2017 to 1.2% (95% CI 1.0–1.5) in 2023 (OR 1.36 (1.03–1.80), *p* = 0.032). Concomitantly, the same statistically significant increasing prevalence was determined for two specific categories; deep incisional (SSI-D) and organ/space (SSI-O) SSIs. At the same period, superficial incisional infections (SSI-I) prevalence showed a statistically significant decrease (Pearson’s Chi-squared test for all SSIs, SSI-S, SSI-D, SSI-O, Fisher’s exact test for small frequencies SSI-Nos).

The proportion of clinically complexed infections (deep incisional and organ-space SSIs combined) rose statistically significant from 48/92 to 77/96; odds ratio (OR) 2.09 (95% CI 1.45–3.01), *p* = 0.001.

In addition, the distribution of the SSI categories between 2017 and 2023 has changed (2 × 4 Chi-squared test χ^2^ = 19.22, df = 3, *p* = 0.00034) showing a different pattern in relative proportions (superficial SSI (SSI-S) decreased from 44.6% to 17.7% (contributing 35% to χ^2^), deep SSI (SSI-D) increased from 28.3% to 49.0% (contributing 50% to χ^2^), organ/space SSI (SSI-O) increased from 23.9% to 31.3% (contributing 14% to χ^2^).

When available, according to the technical protocol in both surveys, risk factors connected to surgical procedures were determined, as presented in Table 6. In 2017, the HCAIs prevalence was higher in patients who underwent NHSN-defined procedures compared to patients without surgical procedures (7.51% vs. 5.13%; χ^2^(1) = 8.30; *p* = 0.00397) and higher in the non-NHSN/minimal intervention group (9.49% vs. 5.13%; χ^2^(1) = 11.00; *p* = 0.00091). In 2023, the HCAIs prevalence was higher in patients with NHSN surgery compared to those without surgery (10.69% vs. 6.56%; χ^2^(1) = 16.47; *p* < 0.0001), while the difference between patients without surgery and the non-NHSN/minimal intervention group was not statistically significant (6.10% vs. 6.56%; *p* = 0.7475).

Comparison between 2017 and 2023 showed a significant increase in HCAIs prevalence in the no-surgery group (+1.43 pp; *p* = 0.0080) and in the NHSN group (+3.18 pp; *p* = 0.020), while the change in the non-NHSN/minimal group showed no statistically significant difference (*p* = 0.103).

### 3.6. Bacterial Causative Agents of Surgical Site Infections

In 2017, 54.3% (50/92) of all reported SSIs had negative microbiological results. By comparison, in 2023, the proportion of negative findings decreased and was 40.6% (39/96). The reasons why the microbiological findings were negative vary in the two observed periods. The majority of negative findings (41/50 (82%)) in 2017 were due to negative culture results in samples taken and processed in microbiology laboratories. In contrast, in 2023, the failure to collect a sample for microbiological processing was present in 76.9% (30/39) cases of negative microbiology results. As presented in Table 7, an analysis of the distribution of microbial agents isolated from SSIs reveals a notable shift between the two study periods. In 2017, the most isolated bacterial strains responsible for SSIs were Gram-positive cocci. However, in 2023, there was a marked change, with Enterobacterales species becoming the predominant group among microbiologically confirmed bacterial pathogens.

This change in the bacterial landscape highlights a transition in the dominant causative agents of SSIs over time, with Gram-positive cocci leading in 2017 and Enterobacterales taking prominence in 2023.

### 3.7. Antibiotic Consumption

Antibiotic use increased between 2017 and 2023, as shown in Table 8. Increasing trends were observed across all indications for antibiotic administration. The prevalence of patients receiving surgical prophylaxis among all patients receiving antibiotics increased significantly over time (χ^2^ = 25.419, df = 1, *p*  < 0.5).

**Figure 2:**
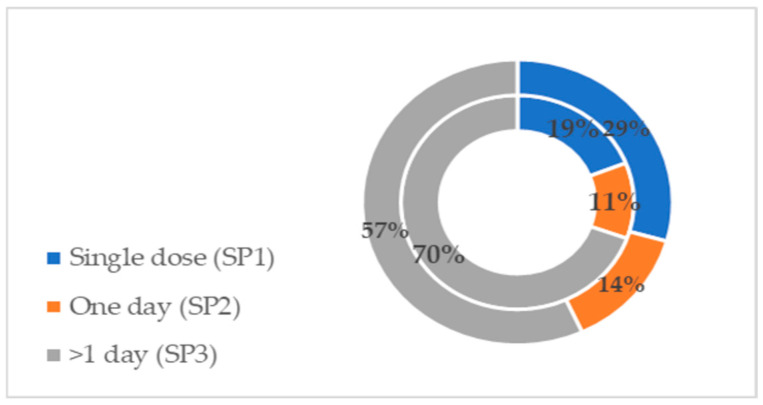
Duration of perioperative antibiotic prophylaxis. Outer circle—2023, inner circle—2017; SP—surgical prophylaxis.

Although the proportion of prolonged PAP (> 24 h) decreased from 70.2% (388/553) to 57.4% (327/570); difference −12.8 pp (95% CI −18.4 to −7.2), it still remains common, with more than half of the hospitalized patients receiving PAP inadequately, as presented in Figure 2.

The stratification of antibiotic consumption in patient groups depending on surgery type (NHSN/non-NHSN category) or absence of surgery is presented in Table 9. This analysis, conducted in part by the ACHs (2017, 2023), revealed that most patients receiving antibiotics in ACHs had undergone a surgical procedure, with a shift in 2023, when that proportion decreased. However, change was observed in surgical procedure categories: 2017 data showed that higher proportions of patients who underwent minimal surgical procedures received antibiotic therapy than patients who underwent major NHSN surgery, whereas in 2023, this changed.

## 4. Discussion

This study provides the first nationwide assessment of epidemiological trends in healthcare-associated infections and antibiotics consumption, specifically focusing on SSI burden and associated antibiotics use in ACHs in Croatia. Furthermore, we have assessed the evolving trends in patterns of microbial pathogens causing SSIs, changes in hospital and surgical departments’ structure indicators, alterations of IPC teams’ structures and prevention processes indicators; consumption of alcohol-based hand rubs and participation in active surveillance.

Although incidence studies allow for finer risk attribution and time-to-event analysis, their higher demands for sufficient human resources, costs and duration, especially in large national studies, often limit their feasibility. In line with EU/EEA practice, standardized point prevalence surveys (PPSs) are a pragmatic tool to track HCAIs and antibiotic use over time, and to benchmark IPC performance and evaluate the impact of prevention interventions across Europe [24,25]. Among HCAIs, SSIs consistently rank among the most prevalent type, highlighting the enduring clinical and economic significance of these infections, as determined by numerous surveillance studies. In addition to insight in the burden of SSIs, participation in active surveillance also may have a positive impact on the reduction in SSIs [26,27,28,29].

Reports of PPSs studies conducted in EU/EEA countries indicate a higher overall HCAI prevalence in 2022–2023 compared to 2016–2017, with significant between-country heterogeneity. The prevalence of patients with at least one HCAI in EU/EEA, as shown in the 2023 report, was 7.1%, and ranged from 3.0% (95% CI 1.5–6.1%) in Latvia to 13.8% (95% CI 8.3–22.1%) in Cyprus, whereas a report from 2017 showed an overall observed prevalence of 5.9% (95% CI 5.8–6.0%) and ranged from 4% in Lithuania (95% CI 2.1–4.0%) to 10.0% (95% CI 8.5–11.6%) in Greece [30]. A similar trend was observed in our study, demonstrating that this increase was mirrored in Croatia: in 2016–2017, its prevalence was below the EU/EEA median (5.8%), whereas in 2022–2023 (6.8%) it was above the median [24,25]. This study adds data on the diverse research worldwide, showing an increase in SSIs prevalence with a marked shift toward deep incisional and organ-space SSIs. This finding may indicate the impact of various different risk factors including reduced adherence of proper hygiene measures over time due to relaxation after pandemics, impact of the decrease in IPC healthcare-workers, more complex patient characteristics such as age, underlying comorbidities and the severity of the disease requiring surgery, or combination of some of these effects.

A concordant increasing trend of deep/organ-space infections after colorectal elective and emergency surgeries was identified by Sun et al. in a 10-year period, and other studies of elective colorectal surgery [31,32]. International guidelines on SSIs prevention provide recommendations of practices in the prevention of SSIs based on different evidence strength and they were published in the period before our first PPS was conducted [33,34].

Aside from hand hygiene guidelines, PAP guidelines and yearly surveillance of HCAIs and antibiotic consumption, recommended by national legislation, Croatia has no other national guidelines and recommendations on other essential components of SSI prevention bundles [35,36,37,38].

Croatian national PAP guidelines were issued in 2010 resulting from multidisciplinary collaboration, recommending one-time administration of antibiotics for most surgical procedures, when necessary [36]. As in other PAP recommendations, there are no procedures that require a prolongation of PAP beyond 24 h after incision. As studies indicate, extending the duration of PAP beyond 24 h may not yield additional benefits in reducing SSIs and may have higher odds of adverse events, such as acute kidney injury, *Clostridioides difficile* infections and simultaneously raising concerns about the effects on antimicrobial resistance [39,40,41]. So far, there has been no research on compliance with PAP guidelines in Croatia. Although dedicated national guidelines are in effect, the prevalence of patients receiving antibiotics for surgical prophylaxis unnecessarily for an extended period (longer than 24 h) was highest among the prevalence of patients receiving surgical prophylaxis for all PAP indications (single dose, one day, >24 h). In comparison to trends on EU/EEA level, a higher prevalence of patients receiving improper prolonged PAP is observed in Croatia (EU/EEA improper prolonged prevalence > 24 h was 2.9%, in 2023 2.8%), furthermore with the increasing trend over time in this study. This in line with the increasing prevalence of antibiotic consumption in ACHs determined for all indications (treatment and surgical prophylaxis).

Besides these findings in prevalence rates among all patients receiving antibiotics, a reduction was observed in the proportion of patients receiving prophylactic antibiotic for longer than 24 h within PAP patients cohort, from 70% in 2017 to 57.4% in 2023. Prolonged surgical antibiotic prophylaxis remains common, affecting more than half of patients requiring PAP. Croatia continues to exceed the EU/EEA median (54% in 2017, following 48% in 2023), indicating persistently inappropriate perioperative antibiotic prophylactic practices [42]. These data call for interventions by multidisciplinary approach, including surgical specialists, anaesthesiologists, IPC specialists, and clinical pharmacists. Corrective measures to improve compliance with existing national PAP guidelines are mandatory.

Shifts of the causative bacterial agents’ patterns towards Gram-negative bacilli align with other research data [43,44,45]. A study by Elgohari et al. showed significant changes in microbial aetiology of SSIs in NHS hospitals in England during past decades, with observed frequencies of *Stapylococcus aureus* isolation decreasing from 41% in 2000 to 16% in 2013, whereas Enterobacterales showed stability in trends from 2000 to 2007, but increased from 2008 as the most frequent pathogens, reaching 25% of cases in 2013 [45]. In contrast to these findings, which are in line with our data, the ECDC 2023 report provides a different pattern of bacterial causative agents isolated in SSIs. According to this report, Gram-positive cocci were the predominant group of isolates, accounting for 44.4% of causative agents, and Enterobacterales family species were isolated in 33.9% of SSIs. The evolving trend of Gram-negative bacilli predominance, as the one observed in this study, together with the emergence of the antimicrobial resistance of Gram-negative bacteria, raises dilemmas on possible changes in preoperative screening, decolonization practices and “one-size-fits all” PAP guidelines [14,18,46,47]. To fully comprehend and tailor future possible changes in PAP, the proportion of unavailable microbiological findings should not be so high. In our 2023 survey, this proportion was 40.9%, which is higher than the overall percentage in EU/EEA countries (27.2%). Sampling, cultivation of isolates and antibiotic susceptibility testing before antibiotic administration in patients with SSIs are crucial, not only as appropriate patient care, but as surveillance of etiological agents, and their antibiotic susceptibility patterns in order to properly tailor antibiotic stewardship [48]. Various factors may account for this trend; lack of resources for sending samples to microbiology laboratories (e.g., decreased funding for microbiological diagnostics, lack of dedicated healthcare-workers) or negative trends in surgical practice (prescribing antibiotics before sampling “in order to fasten the process of healing or to fasten the discharge of the patients”). To change this practice, additional efforts need to be taken in the education of attending surgeons. Further research is needed to fully comprehend the reasons for this negative trend, and to access whether it is stratified differently in specific SSIs categories (superficial or deep-seated SSIs).

An increase in overall prevalence of patients receiving antibiotic therapy over time was also one of the concerning findings of this study. In the analysis of antibiotic consumption stratified according to surgical procedures undergone or not, a vast proportion of patients who did not have surgical procedure during hospitalization received antibiotic treatment. This calls for detailed analysis for antibiotic treatment indications (other HCAIs increasing prevalence, or unrationed use of antibiotics) and indicate the need for antibiotic stewardship across all departments in ACHs.

We have found that between the studied periods, the number of IPC nurses per hospitals and number of ICP medical doctors decreased significantly. Participation of ACHs in SSI surveillance networks decreased without a statistically significant difference. These findings are a concerning trend, since sufficient numbers of qualified IPC personnel and those participating in SSIs surveillance networks have shown to have a positive effect on the decrease in SSIs [27,28,42,49,50].

Rising alcohol-based hand rub consumption in surgical departments is encouraging, but further activities on fostering the establishment of a national network and reengaging hospitals to join surveillance networks, both national and ECDC, are needed.

The main limitations of our study include its design as a prevalence study, and possible case-mix differences in increased deep-seated SSIs between 2017 and 2023. Future research with a case-mix-adjusted analysis is needed to fully comprehend the risk factors for the increase in deep-seated surgical site infections over time. Besides gaps in infection prevention practices, it may reflect patients with multiple comorbidities, higher American Society of Anaesthesiologist (ASA) Physical Status score, and patients from extreme age groups. Additional limitations of the study are a high proportion of non-sampled SSI cases in 2023 (limiting etiologic attribution) and declining of SSI surveillance participation. These factors also can bias comparisons and warrant cautious interpretation. As stated earlier, incidence studies which have a more precise methodology for assessing specific risk factors of SSIs require more resources, which could not be provided equally on the national level due to a lack of HCWs, especially registered IPC nurses in some areas of the country. Furthermore, post-discharge diagnosis of SSIs may have a biased impact on SSIs prevalence surveillance results, and may lead to underestimation of SSIs rates, particularly in implant-related surgical procedures due to SSI-diagnosis time [51,52]. Post-discharge SSIs, including readmissions due to infections after discharge, are generally not covered by prevalence studies’ methodology since these studies record SSIs that are active and present during hospitalization at the time of the survey.

The main study’s strengths include its national scope, standardized ECDC protocols, and repeated measurement. The results of this study provide insight in trends, and challenges of HCAIs, specifically SSIs, showing the need for an evaluation of the implementation preventive measures and their strengthening, although they should not be interpreted as causal effects. The implementation of prevention measures is crucial, since effectiveness varies depending on adherence to established protocols and the specific interventions employed [14,53].

The implications of our study are the recommendations for (i) strict adherence to national perioperative prophylaxis guidelines with audited indicators (indications, timing, dosing, and duration); (ii) strict adherence to SSIs prevention guidelines; (iii) standardized microbiological sampling; (iv) re-engagement in SSIs surveillance networks with benchmarking feedback; and (v) the increase in IPC healthcare-workers.

Further studies using incidence cohorts for high-risk procedures that should confirm trends and identify patient- and procedure-level predictors (age, ASA, comorbidities, duration/complexity) are needed. A regular interrupted time-series should be conducted to test the impact of PAP stewardship and SSIs bundles. Microbiological surveillance should map microbial resistance trends by procedure and possibly guide future PAP updates. Economic evaluations should quantify the cost-effectiveness of preventive measure implementation, as a fundamental part of patient safety in acute care hospitals.

## 5. Conclusions

The prevalence of SSIs in Croatia increased in the period between 2017 and 2023, with a shift towards a higher prevalence of deep-seated surgical site infections. This complies with an increased prevalence of overall healthcare-associated infections.

Antibiotic consumption is higher, with a still high proportion of prolonged perioperative antibiotic prophylaxis, regardless of some improvements in the duration of administration. Mixed results were determined in structure and process indicators; consumption of alcohol-based hand rubs in surgical departments increased, while human resources in infection prevention and participation in active surveillance networks of SSIs decreased. These findings support the need for strengthening the prevention measures package, including ensuring sufficient human resources in infection prevention, which is a fundamental part of patient safety in ACHs, the optimization of perioperative antibiotic prophylaxis, and the inclusion of ACHs in surveillance networks.

## Figures and Tables

**Table 1:** Structure indicators in hospitals during the studied periods.

Structure Indicators	2017	2023
Number of hospital beds/HR mean	458	399
Number of beds in surgical departments/HR mean	128.8	144
Bed occupancy in surgical departments/HR mean	97.2	88.3
Annual number of patient-days in surgical departments	30,387	27,050

HR—Hrvatska (Croatia).

**Table 2:** Prevention indicators.

Prevention Indicators	2017		2023		Abs. Change/pp	Rel. Change	*p*-Value
	Mean% ± SD	Median (IQR)	Mean ± SD	Median (IQR)			
IPC nurses per hospital (N)	2.4 ± 3.0	1.5 (1.0–2.0)	1.8 ± 2.0	1.0 (1.0–2.0)	−0.5	−33.3%	0.041
IPC nurses (N/250 hospital beds)	1.36 ± 2.12	1.0 (0.73–1.23)	1.33 ± 2.24	0.87 (0.71–1.11)	−0.13	−13.0%	0.162
IPC medical doctors (N)	0.73 ± 0.44	1.0 (0.25–1.0)	1.92 ± 4.85	0.5 (0.0–1.0)	−0.5	−50.0%	0.003
IPC medical doctors (N/250 hospital beds)	0.56 ± 0.39	0.61 (0.23–0.78)	1.81 ± 2.51	0.39 (0.0–0.77)	−0.22	−36.1%	0.002
% of ACHs participating in SSIs surveillance networks	0.77		0.68		−9	−11.7%	0.25 *
AHR consumption (L/pt.-days)	22.3 ± 13.9	18.6 (16.1–23.3)	42.3 ± 32.1	34.3 (31.5–42.7)	+15.7	+84.4%	<0.001

N—number; IPC—infection prevention and control; SD—standard deviation; IQR—interquartile range; AHR—alcohol-based hand rub; L/pt.-days—litre per 1000 patient-days; pp—percentage points; abs. change—absolute change; rel. change—relative change; * McNemar’s test for paired binary variable (for other paired prevention indicators, the Wilcoxon Signed-Rank Test was used).

**Table 3:** Point prevalence surveys participants, prevalence of HCAIs in acute care hospitals and prevalence of HCAIs in surgical departments.

		Acute Care Hospitals	Surgical Departments
HR-PPS-1 (2017)	N of study participants	10,466	2985
	R%	100%	28.50%
	N HCAI	551	151
	% HCAI	5.30% (95% CI 4.8–5.7)	5.10%
HR-PPS-2 (2023)	N of study participants	8066	2383
	R%	1	29.50%
	N HCAI	643	160
	% HCAI	7.20% (95% CI 6.6–7.8)	6.70%
Pearson’s Chi-squared test	Difference	2.71%	1.65%
	95% CI	1.98–3.44%	0.38–2.93%
	*p*-value	9.93 × 10^−14^	0.0099

HR-PPS-1—HR (Croatia) Point Prevalence Survey-1; HR-PPS-2—HR (Croatia) Point Prevalence Survey-2; N—number; R—relative frequency of patients participating in the surveys; HCAIs—healthcare-associated infections.

**Table 4:** Site-specific infections 2017–2023.

	2017		2023			
	N of P ^1^	R% ^2^	N of P ^1^	R% ^2^	pp%	*p*-Value *
Urinary tract infections	163	27.9%	176	27.4%	0.21	0.8329
Pneumonia	114	19.5%	136	21.2%	−0.71	0.4789
Surgical site infections	92	15.8%	96	14.9%	0.4	0.6892
Bloodstream infections	59	10.1%	85	13.2%	−1.69	0.0903
Catheter-related infections	8	1.4%	5	0.8%	1.01	0.3116
GI-CDI (*C. difficile* infection)	28	4.8%	43	6.7%	−1.42	0.1561

^1^ N of P—number of patients; ^2^ R%—relative frequency (%); pp—percentage points; * Pearson’s Chi-squared test was used for the calculation of *p*-value.

**Table 5:** Surgical site infection distribution according to the affected tissue depth.

	2017				2023			
	N of P	Prevalence (95% CI)	R%	N of P	Prevalence (95% CI)	R%	OR	*p*-Value
SSI, all	92	0.9% (0.7–1.1)	15.8%	96	1.2% (1.0–1.5)	14.9%	1.36 (1.03–1.80)	0.032
SSI-S	41	0.4% (0.3–0.5)	7.0%	17	0.2% (0.1–0.3)	2.6%	0.53 (0.30–0.95)	0.028
SSI-D	26	0.2% (0.2–0.4)	4.5%	47	0.6% (0.4–0.8)	7.3%	2.37 (1.463.84)	0.0031
SSI-O	22	0.2% (0.1–0.3)	3.8%	30	0.4% (0.3–0.5)	4.7%	1.78 (1.03–3.09)	0.038
SSI-Nos	3	0.0% (0.0–0.1)	0.5%	2	0.0% (0.0–0.1)	0.3%	0.87 (0.14–5.22)	1.0
2 × 4 Chi-squared test	χ^2^ = 19.22, df = 3, *p* = 0.00034							

SSIs—surgical site infections; SSI-S—superficial incisional; SSI-D—deep incisional SSI; SSI-O—organ/space SSI; SSI Nos—category not specified/unknown SSI; N of P—number of patients; R—relative frequency.

**Table 6:** Distribution of PPSs participants and HCAIs depending on surgical procedures undertaken.

Surgery Category	No Surgery	NHSN Surgery	Non-NHSN/Minimal Surgery	Missing/Unknown
N of patients (R%)				
2017	4563 (78.6%)	919 (15.8%)	316 (5.4%)	8 (0.1%)
2023	3127 (72.5%)	842 (19.5%)	344 (8.0%)	2 (0.0%)
N of HCAIs (R%)				
2017	234 (5.1%)	69 (7.5%)	30 (9.5%)	0 (0.0%)
2023	205 (6.6%)	90 (10.7%)	21 (6.1%)	0 (0.0%)
* Pearson’s Chi-squared test 2017–2023	1.43	3.81	−3.39	
Difference 95% CI	0.35–2.51	0.49–5.87	−7.49–0.72	
*p-* value	0.008	0.02	0.103	

N—number; R—relative frequency; HCAIs—healthcare-associated infections; NHSN—National Healthcare Safety Net Work Code; * Pearson’s Chi-squared test between HCAI prevalence 2017–2023 in different NHSN categories.

**Table 7:** Distribution of SSIs microbial causative agents.

Microorganism	2017 N (R%)	2023 N (R%)	*p*-Value
Total SSI with microorganisms	42 (45.7)	57 (59.4)	—
Gram-positive cocci	29 (44.6)	28 (33.7)	0.0756
*Staphylococcus aureus*	8 (12.3)	6 (7.2)	—
Coagulase-negative *Staphylococcus* spp.	5 (7.7)	8 (9.6)	—
*Streptococcus* spp.	3 (4.6)	1 (1.2)	—
*Enterococcus* spp.	12 (18.5)	13 (15.7)	—
Enterobacterales	17 (26.2)	35 (42.2)	0.0633
*Enterobacter* spp.	3 (4.6)	3 (3.6)	—
Escherichia coli	5 (7.7)	6 (7.2)	—
*Klebsiella* spp.	5 (7.7)	13 (15.7)	—
*Proteus* spp.	4 (6.2)	6 (7.2)	—
Other Enterobacterales	0 (0.0)	3 (3.6)	—
Gram-negative, non-Enterobacterales	17 (26.2)	13 (15.7)	0.0950
*Acinetobacter* spp.	7 (10.8)	6 (7.2)	—
*Pseudomonas aeruginosa*	9 (13.8)	5 (6.0)	—
*Stenotrophomonas maltophilia*	0 (0.0)	1 (1.2)	—
Anaerobic bacilli	0 (0.0)	1 (1.2)	—
Fungi	2 (3.1)	5 (6.0)	—
*Candida* spp.	2 (3.1)	5 (6.0)	—
*Aspergillus* spp.	0 (0.0)	0 (0.0)	—
Negative microbiology findings	50 (54.3)	39 (40.6)	0.0823
Microorganisms not identified	41 (44.6)	6 (6.3)	—
Examination not done	2 (2.2)	3 (3.1)	—
Sterile examination	5 (5.4)	0 (0.0)	—
Not available	2 (2.2)	30 (31.3)	—

SSI—surgical site infection; N—number; R—relative frequency; Data are presented as counts and percentages relative to the total number of SSI cases per year; *p*-values calculated using the Pearson’s Chi-squared test (or Fisher’s exact test where applicable); “—” indicates; *p*-value not calculated due to low counts or descriptive comparison only.

**Table 8:** Administered antibiotics; indications, prevalence and proportions of patients receiving antibiotic therapy.

	2017			2023				
Indication	N of P	P% (95% CI)	N of a	R%	N of P	P% (95% CI)	N of a	R%
Total	3263	31.2% (30.3–32.1)	4714	100%	3237	40.1% (39.1–41.2)	4933	100%
Treatment intention	2136	20.4% (19.6–21.2)	3077	65.3%	1996	24.7% (23.8–25.7)	3108	63%
Community infection (CI)	1501	14.3% (13.7–15.0)	2133	45.2%	1372	17.0% (16.2–17.8)	2066	41.9%
HCAI	533	5.1% (4.7–5.5)	754	16%	548	6.8% (6.3–7.4)	898	18.2%
Surgical prophylaxis	553	5.3% (4.9–5.7)	710	15.1%	570	7.1% (6.5–7.6)	742	15%
Single dose (SP1)	106	1.0% (0.8–1.2)	124	2.6%	169	2.1% (1.8–2.4)	189	3.8%
One day (SP2)	64	0.6% (0.5–0.8)	76	1.6%	79	1.0% (0.8–1.2)	99	2%
>1 day (SP3)	388	3.7% (3.4–4.1)	510	10.8%	327	4.1% (3.6–4.5)	454	9.2%

N of P—number of patients receiving >1 antibiotic; P% (95% CI)—prevalence of patients receiving >1 antibiotic; N of a—number of antibiotics; R—relative frequency of administrated antibiotics for specific indication; HCAI—healthcare-associated infection; SP—surgical prophylaxis.

**Table 9:** Patients receiving antibiotic therapy depending on undertaking of surgical procedure.

Surgery Category	No Surgery	NHSN Surgery	Non-NHSN/Minimal Surgery
	N of P/R (%)	N of P/R (%)	N of P/R (%)
2017	1379 (30.2%)	464 (50.5%)	175 (55.4%)
2023	1328 (42.5%)	534 (63.4%)	189 (54.9%)

N of P—number of patients; R—relative frequency of patients among all patients in the category; NHSN—National Healthcare Safety Network.

## Data Availability

The original contributions presented in the study are included in the article, further inquiries can be directed to the corresponding author.
